# Case Report: Severe Adolescent Major Depressive Syndrome Turns Out to Be an Unusual Case of Anti-NMDA Receptor Encephalitis

**DOI:** 10.3389/fpsyt.2021.679996

**Published:** 2021-05-25

**Authors:** Alexander Moldavski, Holger Wenz, Bettina E. Lange, Cathrin Rohleder, F. Markus Leweke

**Affiliations:** ^1^Department of Psychiatry and Psychotherapy, Central Institute of Mental Health, Medical Faculty Mannheim, Heidelberg University, Mannheim, Germany; ^2^Department of Neuroradiology, Medical Faculty Mannheim, Heidelberg University, Mannheim, Germany; ^3^Brain and Mind Centre, Faculty of Medicine and Health, The University of Sydney, Sydney, NSW, Australia; ^4^Sydney Local Health District, NSW Health, Sydney, NSW, Australia

**Keywords:** autoimmune encephalitis, anti-neuronal autoantibodies, depression, isolated psychiatric presentation, young adult, cerebrospinal fluid

## Abstract

Anti-N-methyl-D-aspartate receptor (NMDAR) encephalitis is a neuroinflammatory condition mediated by autoantibodies against the GluN1 subunit of the receptor. Clinically, it is characterized by a complex neuropsychiatric presentation with rapidly progressive psychiatric symptoms, cognitive deficits, seizures, and abnormal movements. Isolated psychiatric manifestations of anti-NMDAR encephalitis are rare and usually dominated by psychotic symptoms. We present a case of an 18-year-old female high school student—without a previous history of psychiatric disorders—with a rapid onset severe depressive syndrome. Surprisingly, we found pleocytosis and anti-NMDAR autoantibodies in the cerebrospinal fluid (CSF), despite an otherwise unremarkable diagnostic workup, including blood test, clinical examination, and cranial magnetic resonance imaging (MRI). After intravenous immunoglobulins treatment, a complete remission of the initial symptoms was observed. In a follow-up 5 years later, the young woman did not experience any relapse or sequelae. Anti-NMDAR encephalitis can present in rare cases as an organic disorder with major depressive symptoms without distinct concomitant psychotic or neurological symptoms. A clinical presentation such as a rapid onset of symptoms, distinct disturbance in the thought process, restlessness, and cognitive deficits should prompt screening for NMDAR- and other neural autoantibodies to rule out this rare but debilitating pathology.

## Introduction

Anti-N-methyl-D-aspartate receptor (NMDAR) encephalitis is an autoantibody (AAb)-mediated immune disease that presents as a complex transdiagnostic neuropsychiatric syndrome with rapidly progressive psychiatric symptoms, cognitive deficits, seizures, abnormal movements, and coma in severe cases. In women, almost 40% of the cases are associated with an ovarian teratoma (1, 2).

NMDARs are glutamate-gated non-selective cation channels that are expressed on the majority of excitatory synapses and play a crucial role in development, synaptic plasticity, and a variety of neuropsychiatric pathologies (3, 4).

Although anti-NMDAR encephalitis is a rare condition (estimated incidence of 1.5 per million populations a year), its clinical presentation, reminiscent of schizophreniform illness, makes it an important differential diagnosis in psychiatry due to an effective causal treatment option (1). In addition, its character as a model disease for two major hypotheses of the pathophysiology of mental disorders, namely the glutamatergic and immune hypothesis, underlines the importance of this disorder (4–6).

One study suggested that acutely ill patients with an initial schizophrenia diagnosis also without evidence of encephalitis show an increased prevalence of NMDAR AAbs in serum (7). The relevance of serum NMDAR AAbs is still unclear since only AAbs in cerebrospinal fluid (CSF) appear to be pathognomonic (8, 9). However, NMDAR AAbs were neither detected in CSF of 124 individuals with schizophrenia spectrum disorders (10) nor in CSF and serum of first-episode schizophrenic psychosis patients (FEP, *n* = 103), clinical high risk for psychosis individuals (CHR, *n* = 47), and healthy volunteers (HV, *n* = 40) (11).

In a large observational cohort of 571 individuals with diagnosed anti-NMDAR encephalitis, only 4% of the cases presented with an isolated psychiatric manifestation and <1% in the initial episode of the disorder (1, 12). Notably, the psychopathology of those cases is dominated by psychotic, cognitive, and catatonic symptoms (12–14). To our knowledge, there are only two case reports in the literature of anti-NMDAR encephalitis, which mimic a major depressive episode with psychotic symptoms (15, 16). Here we report the first case of a patient with anti-NMDAR encephalitis, which presents as a major depressive episode without distinct psychotic symptoms like delusion, hallucination, or catatonia, as well as without any neurological signs or symptoms.

## Case Description

**Figure 1:**
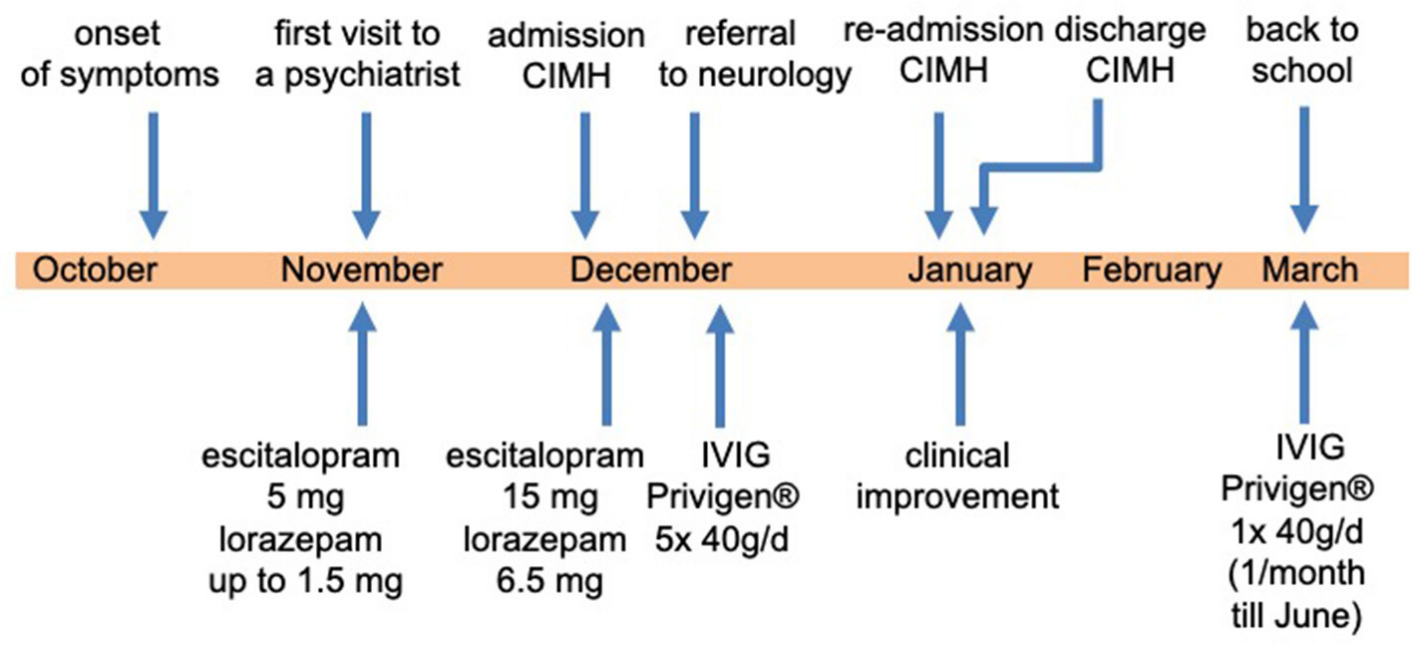
Relevant data from this episode of care organized as a timeline.

An 18-year-old female high school student without a history of psychiatric disorder was admitted to our day-care clinic at the Central Institute of Mental Health (CIMH), Medical Faculty Mannheim, Heidelberg University (Figure 1). On admission, she complained that she “is not the same person, [she] used to be before.” Being a very ambitious, self-confident student and a competitive athlete, she started to become increasingly insecure and was confronted with unusual self-doubts. During her last school vacation (~6 weeks before admission), the relationship with her boyfriend ended abruptly.

Following her return to school after vacation, she developed unfamiliar exam nerves. She was sitting in front of her assignments and having the impression that her “thoughts were blocked.” She was continually ruminating over her condition and feared that she would never be “the same” again. She showed depressed mood and markedly diminished interest in almost all activities as well as substantially reduced drive and social interaction, which was associated with the concern that “people might notice, how bad [her] condition is.” This culminated in an inability to leave the house at all. Occasionally, she had the impression that people were talking about her condition. It occurred briefly a few times prior to admission, and upon request, she considered it highly unlikely that this happened in reality.

On admission, we saw a well-dressed and well-groomed young woman. During the interview, she was slightly restless and presented a cautious and mildly suspicious attitude toward the examiner and a marked ambivalence regarding the treatment. Nevertheless, she remained cooperative and friendly most of the time. She was in a depressed mood, and her affect showed no modulation and reactivity throughout the interview. Markedly diminished interest in almost all activities and substantially reduced drive and social interaction were reported. The flow of thought was slow and thought blocking was rarely observed and self-experienced. The thought process itself remained generally coherent. Thought pressure was reported. Besides some occasional unstable ideas of reference, the thought content was unremarkable, with no evidence of delusions, hallucination, or self-disorder. There was also no evidence of obsessive-compulsive symptoms. She was oriented and showed a marked reduction in attention and concentration. Short-term memory showed mild disturbances, while long-term memory was unaffected. Reduced appetite, libido, and sleep disturbances were reported. The young woman clearly and consistently denied any form of suicidal ideation.

There was neither a history of drug abuse nor of a previous episode of a mental disorder. However, the young woman reported a history of mental health issues in her family. Her father and her aunt (paternal) suffered from a major depressive episode; her grandfather (paternal) had post-stroke depression. As a preexisting medical condition, hypothyroidism was stated.

**Figure 2:**
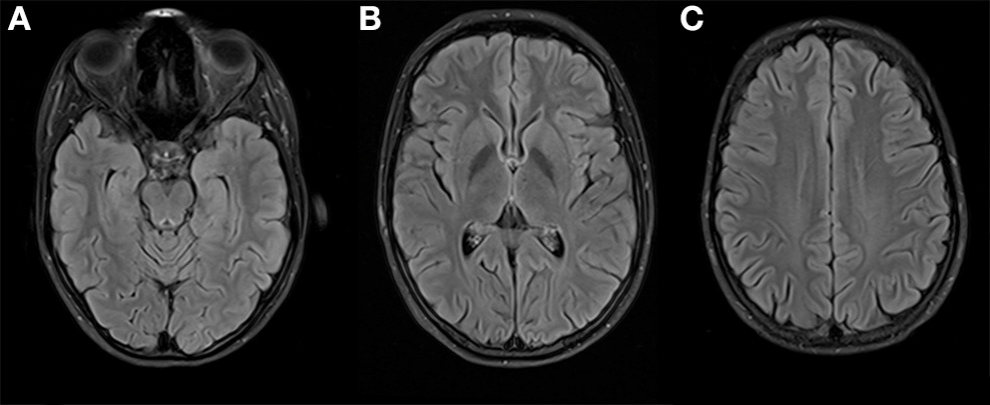
Brain imaging at 3 Tesla MRI (T2 FLAIR weighted) on three different levels **(A–C)** shows no suspicious signal alterations, especially on both mesial temporal lobes **(A)** and insula **(B)**.

We diagnosed severe major depression (MDD) according to the ICD-10. Based on the aforementioned mental status examination, some attenuated positive symptoms (marked suspicion and unstable ideas of reference) and basic symptoms (thought blockade and thought pressure) according to the Early Recognition Inventory (ERIraos) (17) could be detected. However, those symptoms never reached the threshold for psychosis, and severe depressive symptoms dominated the overall clinical picture (18). Results of further clinical examinations and initial blood tests were within the normal range, besides iatrogenic hyperthyroidism. The initial cranial magnetic resonance imaging (MRI) revealed no pathological findings. We continued the already initiated administration of escitalopram (5 mg) and lorazepam (1.5 mg/d) and increased the dose of escitalopram stepwise to 15 mg and lorazepam up to 6.5 mg daily. We further reduced the initial dose of 150 μg of L-thyroxin to 100 μg due to the increased thyroid hormone levels. The young woman showed a marked improvement after the administration of lorazepam, especially regarding the thought process disturbances and cognitive deficits. The young woman consented to a lumbar puncture, revealing a subtle pleocytosis and anti-NMDAR AAbs (titer 1:4) in CSF. She was subsequently referred to the Department of Neurology of the University Medicine Mannheim, Faculty Mannheim of Heidelberg University (UMM), where some diagnostic procedures were repeated. The follow-up MRI at UMM did not reveal pathological findings (Figure 2). Several consecutive EEG recordings were within the normal range. The repeated CSF examination confirmed the presence of anti-NMDAR AAbs (titer 1:8) and pleocytosis (eight cells). Notably, no anti-NMDAR AAbs were detected in serum. A further comprehensive neuroimmunological, viral, and microbiological examination did not result in any additional pathological findings. A whole-body MRI did not reveal any potential origin of a possible para-neoplastic genesis of the underlying condition.

**Figure 3:**
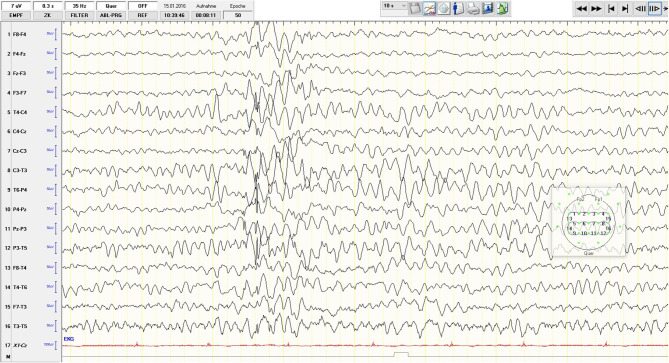
Sixteen-channel EEG recording, low-pass filter 35 Hz: desynchronize alpha-rhythm (7–10 Hz) with a high amplitude (60–150 μV); generalized irregular spikes and sharp waves and occasional theta-rhythm activity.

Subsequently, the young woman received a 5-day course of intravenous immunoglobulins treatment (Privigen® 40 g/d; 200 mg in total). Two weeks after the intravenous immunoglobulin administration, the patient was referred back to our psychiatric department at CIMH. After readmission, we saw a significant improvement in the clinical state, although marked depressive and cognitive symptoms remained. We repeated an EEG during the tapering off phase of the initially high dose of lorazepam. In contrast to the previous regular EEG recordings, an intermittent epileptogenic activity was detected (generalized irregular spikes and sharp waves, additionally desynchronize alpha-rhythm (7–10 Hz) with a high amplitude (60–150 μV), occasionally theta-rhythm) (Figure 3). Consequently, an anticonvulsive treatment with lamotrigine (initially 25 mg/d) was started. The patient and her parents decided to discontinue the psychiatric treatment at CIMH and sought the second opinion of a neuroimmunology specialist at the Department of Neurology, University Hospital of Heidelberg. Her condition was continuously improving, and approximately 7 weeks after her discharge from CIMH, she was able to resume her studies. From March until July 2016, she received monthly immunoglobulin treatments as an outpatient of the Department of Neurology, University Hospital of Heidelberg. She recovered completely without any sequelae and successfully completed her high school certificate (Abitur) the following year. At a follow-up 5 years post-treatment, she was still in good health without any relapse of the disease so far.

## Discussion

Here we present the third published case of anti-NMDA receptor encephalitis, which resembles a major depression without any initial neurological signs or symptoms. In contrast to the other two cases, a 16-year-old girl (15) and a 52-year-old woman (16), our patient did not show any distinct concomitant psychotic symptoms, other than some attenuated positive symptoms and basic symptoms according to a sensitive early recognition scale for psychosis, ERIraos. Although the clinical presentation was dominated by core symptoms of depression and familiar features like preceding psychosocial stress factors (break up of a relation, graduation year), there were also some apparent atypical aspects. The rapid onset of symptoms (within 1–2 weeks), unfamiliar suspicion, unstable ideas of reference, thought blockade, and thought pressure are sometimes observed in early depressive episodes in adolescents but may also represent early warning signs of clinical high risk for psychosis. The latter is often associated with depressive syndromes (19). Another peculiar aspect of the case was the requirement of high doses of lorazepam (up to 6.5 mg a day), which might have masked the epileptiform activity in the initial EEGs. In contrast to brain imaging, which is relatively unspecific, the sensitivity of abnormal EEG patterns is high (96%) in anti-NMDAR encephalitis (20). Extreme Delta Brush (EDB), a pathognomonic EEG finding, is described in 30–58% of abnormal EEGs. In addition, Generalized Rhythmic Delta Activity (GRDA) (50%), spikes (62%), and slow-waves (100%) can also frequently be detected (21, 22). The intensive application of benzodiazepines might be one possible explanation for the rare oligosymtomatic presentation of the disorder in the presented case, as it could have masked some neurological symptoms by suppressing seizures. Also, the low AAb titer (1:4 in the initial lumbar puncture, 1:8 prior to the application of the first course of immunoglobulin, and 1:2 three weeks after the first treatment) may have contributed to the atypical anti-NMDAR encephalitis since low titers are known to be associated with a benign course of the disease (8).

We assume that despite the low titer, there was a causal relationship between the presented condition and the detection of anti-NMDAR AAbs. Several lines of evidence are supporting this assumption. First, in contrast to serum, the presence of NMDAR-AAbs in CSF is highly specific for anti-NMDAR encephalitis (10, 11, 23). Second, high dose lorazepam may have prevented the neurological manifestation of the disorder from evolving, and third, the patient responded well to the immune globulin treatment. Given the anxiolytic and antidepressant effects of NMDAR antagonists like ketamine, it appears counterintuitive that anti-NMDAR AAbs mediate a depressogenic effect (24). On the other hand, NMDAR activation may lead to opposite effects depending on the subcellular localization and the subunit composition of the receptor (3, 25).

## Conclusions

This case highlights the importance of recognizing even subtle atypical patterns of psychiatric manifestations, acquiring CSF for further analysis, and performing comprehensive neurological assessments while taking the effects of already administered medication into account to detect autoimmune-mediated encephalitides reliably.

Recent international guidelines focusing on autoimmune encephalitis as a differential diagnosis of schizophreniform psychosis already recommend EEG and MRI diagnostics, lumbar puncture, and serum and CSF diagnostics (6, 17, 26, 27). However, this case shows that anti-NMDAR encephalitis can present in rare cases as an organic disorder with major depressive symptoms without distinct concomitant psychotic and neurological symptoms. Thus, applying a lower threshold for an autoimmune diagnostic in psychiatry might reveal more anti-NMDAR encephalitis cases and help us understand the link between the psychiatric symptoms and features of the immune response like antibody titer, epitope specificity, and the class of antibodies.

Notably, early identification and initiation of treatment can help to circumvent an unfavorable outcome of this disorder.

## Patients Perspective

“Soon after the onset of the disease, I realized that something was wrong with my mind.

I had the impression that every single thought was being “blocked” even before I was able to reach any conclusion. In addition, I was suffering from constant rumination, which led to severe sleeping problems. It was devastating for me to realize that I could not perform reasonably in school anymore.

Despite these symptoms, I was still aware of everything in my surroundings and could recall every situation during my treatment on the ward. Most of the time, I felt very tired, probably due to a high dose of Lorazepam (Tavor®). To the big surprise of my physicians, I was still able to read books all day long, apprehend and recall their content.

After my discharge from the psychiatric ward [three weeks after the immunoglobulin treatment], I felt a gradual improvement in my condition every day. Retrospectively, I believe that the psychiatric hospital was the wrong place for me since I was not suffering from a mental disorder.

Ten weeks after the first immunoglobulin treatment, I returned to school and was soon able to resume my athletics training (4–5 times a week). I successfully passed my graduation exam (Abitur) the following year and enrolled in college thereafter. After successfully graduating, I am now working full-time as a training officer. I have never experienced these symptoms anymore again.”

## Data Availability Statement

The original contributions presented in the study are included in the article/supplementary material, further inquiries can be directed to the corresponding author/s.

## Ethics Statement

Written informed consent was obtained from the individual(s) for the publication of any potentially identifiable images or data included in this article.

## Author Contributions

AM, BEL, and FML were involved in the patient's treatment at the Central Institute of Mental Health (CIMH), Mannheim. FML initiated the publication process. AM wrote the manuscript. FML and CR revised the intellectual content of the manuscript. HW provided and analyzed the MRI data. All authors contributed to the final manuscript preparation and have read and approved the final manuscript.

## Conflict of Interest

FML is a shareholder of curantis UG (ltd.) and receives research support from the German Federal Ministry of Education and Research (BMBF), the Moyira Elizabeth Vine Fund for Research into Schizophrenia, and the DVCR research fund of the University of Sydney. The remaining authors declare that the research was conducted in the absence of any commercial or financial relationships that could be construed as a potential conflict of interest.
